# Identification of a novel gene signature in second-trimester amniotic fluid for the prediction of preterm birth

**DOI:** 10.1038/s41598-021-04709-3

**Published:** 2022-03-31

**Authors:** Min-A Kim, Eun-Ju Lee, Wookyeom Yang, Ha-Yeon Shin, Young-Han Kim, Jae-Hoon Kim

**Affiliations:** 1 Department of Obstetrics and Gynecology, Gangnam Severance Hospital, Institute of Women’s Life Medical Science, Yonsei University College of Medicine, Seoul, Korea; 2 Department of Obstetrics and Gynecology, Severance Hospital, Institute of Women’s Life Medical Science, Yonsei University College of Medicine, 50-1 Yonsei-ro, Seodaemun-gu, Seoul, 03722 Korea

**Keywords:** Predictive markers, Preventive medicine

## Abstract

Preterm birth affects approximately 5% to 7% of live births worldwide and is the leading cause of neonatal morbidity and mortality. Amniotic fluid supernatant (AFS) contains abundant cell-free nucleic acids (cfNAs) that can provide genetic information associated with pregnancy complications. In the current study, cfNAs of AFS in the early second-trimester before the onset of symptoms of preterm birth were analyzed, and we compared gene expression levels between spontaneous preterm birth (n = 5) and term birth (n = 5) groups using sequencing analysis. Differential expression analyses detected 24 genes with increased and 6 genes with decreased expression in the preterm birth group compared to term birth. Upregulated expressions of RDH14, ZNF572, VOPP1, SERPINA12, and TCF15 were validated in an extended AFS sample by quantitative PCR (preterm birth group, n = 21; term birth group, n = 40). Five candidate genes displayed a significant increase in mRNA expression in immortalized trophoblast HTR-8/SVneo cell with H_2_O_2_ treatment. Moreover, the expression of five candidate genes was increased to more than twofold by pretreatment with lipopolysaccharide in HTR-8/SVneo cells. Changes in gene expression between preterm birth and term birth is strongly correlated with oxidative stress and infection during pregnancy. Specific expression patterns of genes could be used as potential markers for the early identification of women at risk of having a spontaneous preterm birth.

## Introduction

Preterm birth, defined as birth before the completion of 37 weeks of gestation, is the leading cause of neonatal morbidity and mortality. Despite recent advances in neonatal care, the burden of disease in preterm birth remains significant, increasing the risk of developmental and medical disabilities^1^. Spontaneous preterm birth constitutes a complex and multi-factorial phenotype accompanied by numerous gestational tissues to induce parturition and is the result of an interaction of both genetic and environmental factors^2–5^. However, the genetic and molecular characteristics of preterm birth remain elusive and preventive strategies have not yet been established. Amniotic fluid (AF) contains nutrients and growth factors for fetal development and is a source of cells for the prenatal diagnosis of chromosomal abnormalities and fetal infections, as well as for the determination of fetal lung maturity^6,7^. While the cells present in the AF are utilized in these diagnostic methods, the remaining AF supernatant (AFS) is usually discarded. However, the AFS contains abundant cell-free nucleic acids (cfNAs), a uniquely dispersed form of genetic material. CfNAs may be derived from active cellular secretions via exosomes and shedding vesicles or microparticles through apoptosis or necroptosis^8,9^.

AF-derived cfNA is of relatively fetal origin and is less likely to be contaminated with maternal-derived nucleic acids^10–14^. Moreover, the AF directly contacts the placenta and may also include cfNAs originating from placental tissues^15^. CfNAs can serve as intermediate messengers to convey genetic information and cellular signals, which affect various cellular responses such as oxidative stress and immune response^9^. Recently, molecular studies using next-generation sequencing (NGS) have suggested that cfNAs obtained from AFS could serve as a good diagnostic tool for the detection of abnormal fetal conditions such as fetal aneuploidies^16–19^, fetal growth restriction^20,21^, twin to twin transfusion syndrome^22^, and altered molecular pathways^23,24^. However, despite substantial research on cfNAs in fetal development, the correlation between AFS-derived cfNAs and preterm birth is still elusive.

The aim of this study is to characterize the gene expression signatures related to a spontaneous preterm birth from AFS during second-trimester pregnancy and to reveal the biological information about genes involved in the pathogenesis of preterm birth for early diagnosis and appropriate treatment. We hypothesized that the cfNAs from AFS could provide real-time information on spontaneous preterm birth along with nucleic acid from amniocytes.

## Results

### Clinical characteristics of the study population

We obtained 74 AF samples from pregnant women at 16 to 19 weeks of gestation. As a result of amniocentesis in all study populations, no patients were diagnosed with chromosomal abnormalities, including Down syndrome. Among them, 24 women had a preterm delivery, and 50 delivered at term. The average amounts of total nucleic acid in the spontaneous preterm birth and term birth groups were 219.2 ± 102.1 ng and 151.3 ± 69.27 ng, respectively, which were significantly different (P = 0.0012). To pursue sequencing analysis, we paired cases and controls based on the amount of total nucleic acid and selected 5 of the 21 preterm birth and 5 of the 40 term birth samples. The demographic and clinical characteristics of the study population are presented in Supplementary Table 1. There were no significant differences in maternal age (35.2 ± 2.9 vs. 37.0 ± 4.0, years), body mass index (22.8 ± 3.4 vs. 19.7 ± 2.1, kg/m^2^), or gestational age at amniocentesis (17.3 ± 0.8 vs. 17.1 ± 0.9, weeks of gestation) between preterm birth and term birth groups, whereas a significant difference was observed between both groups for gestational age at delivery (35.5 ± 1.3 vs. 40.3 ± 0.3, weeks of gestation).

### Identification of differentially expressed genes between preterm birth and term birth by sequencing analysis

**Figure 1:**
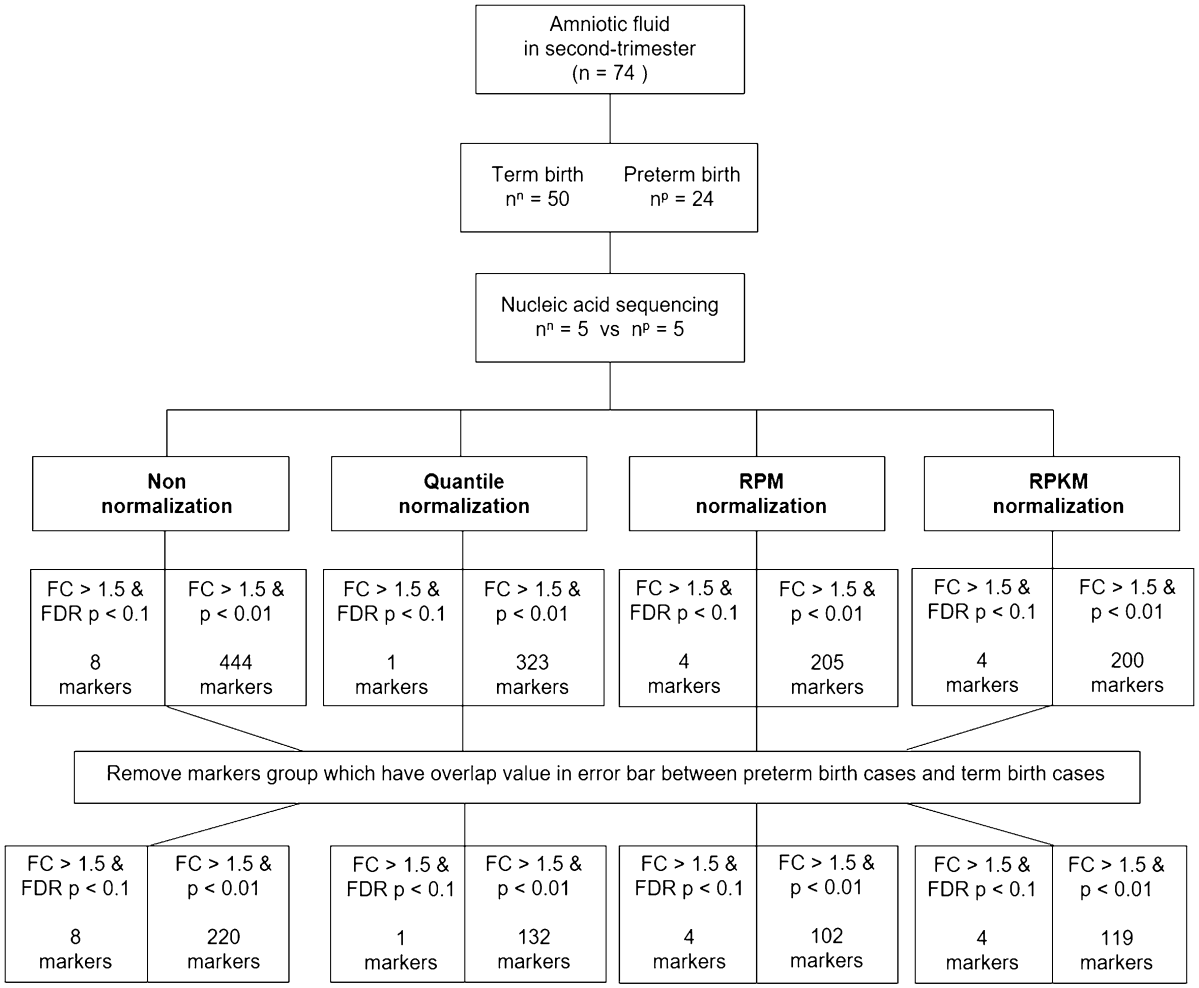
Flow chart of normalization procedures and differentially expressed genes in each normalization method. *FDR* false discovery rate, *FC* fold change. The uppercase letter P is used for preterm birth and N for normal term birth.

**Figure 2:**
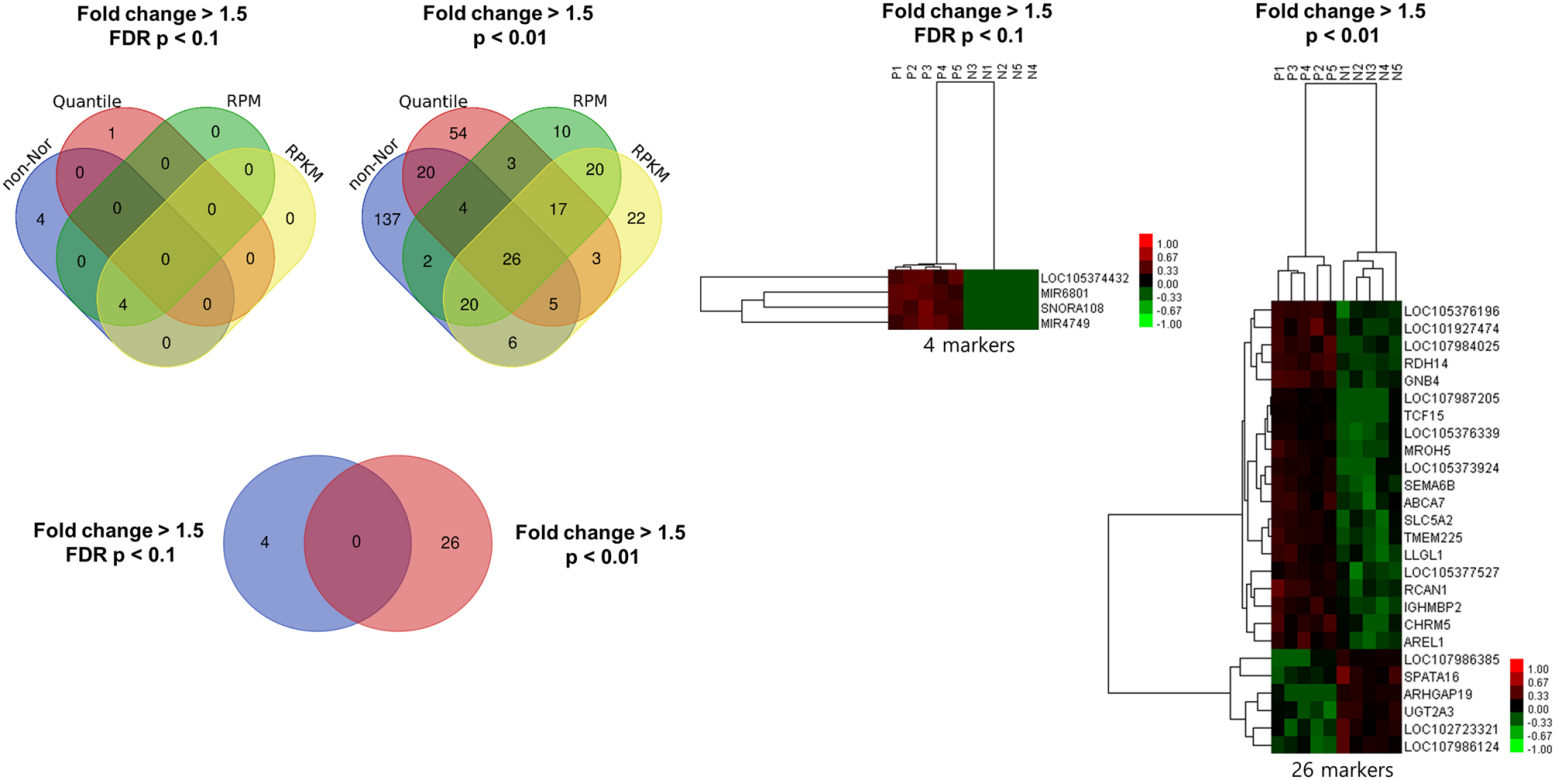
Venn diagram and heat-map comparison of differentially expressed genes from non-normalization, quantile, RPM, RPKM normalization methods.

To examine the gene expression signatures in women destined to have a preterm birth, we performed nucleic acid sequencing on second-trimester AF samples and determined the differentially expressed genes (DEGs) between the preterm and term birth groups. The body fluid samples constantly change components such as RNA/DNA/protein according to intrinsic and extrinsic environmental conditions. Although various normalization approaches have been proposed, there is no standardized manner. Therefore, we applied three normalization methods, i.e., Quantile, reads per million (RPM), and reads per kilobase million (RPKM) normalization, in addition towhil the non-normalization method. Figure 1 shows the flow chart of normalization procedures for each of the five AFS samples in preterm birth and term birth groups and the number of DEGs in each normalization method. It showed quite similar results of overlapping DEGs in the different normalization methods. Gene expression was considered significant if the false discovery rate (FDR) P-value was < 0.1 or P-value < 0.01 with fold change (FC) > 1.5. We obtained 30 genes that were significantly differentially expressed (DE) between the preterm birth and term birth groups. Among these genes, a total of four genes, MIR6801, SNORA108, MIR4749, and LOC105374432, were most commonly DE according to three methods applied with FC > 1.5 and FDR P-value < 0.1, excepted quantile method, and all four genes were upregulated. A total of 26 genes were DE with FC > 1.5 and P-value < 0.01. Among the 26 DEGs, 20 were upregulated, and 6 were downregulated in the preterm birth relative to the term birth groups. We generated a heat map of the DEGs for preterm birth samples with respect to term birth samples. We found unique gene expression profiles from each of these four different normalization methods by pairwise comparisons. The overlap in the results from each normalization method is illustrated in Fig. 2. The full list of DEGs detected is shown in Supplementary Table 2, and the gene expression levels of the top-ranked genes based on a cutoff of FC > 1.5 (FDR p-value < 0.1 or p-value < 0.01) were shown in Supplementary Fig. 1.

**Figure 3:**
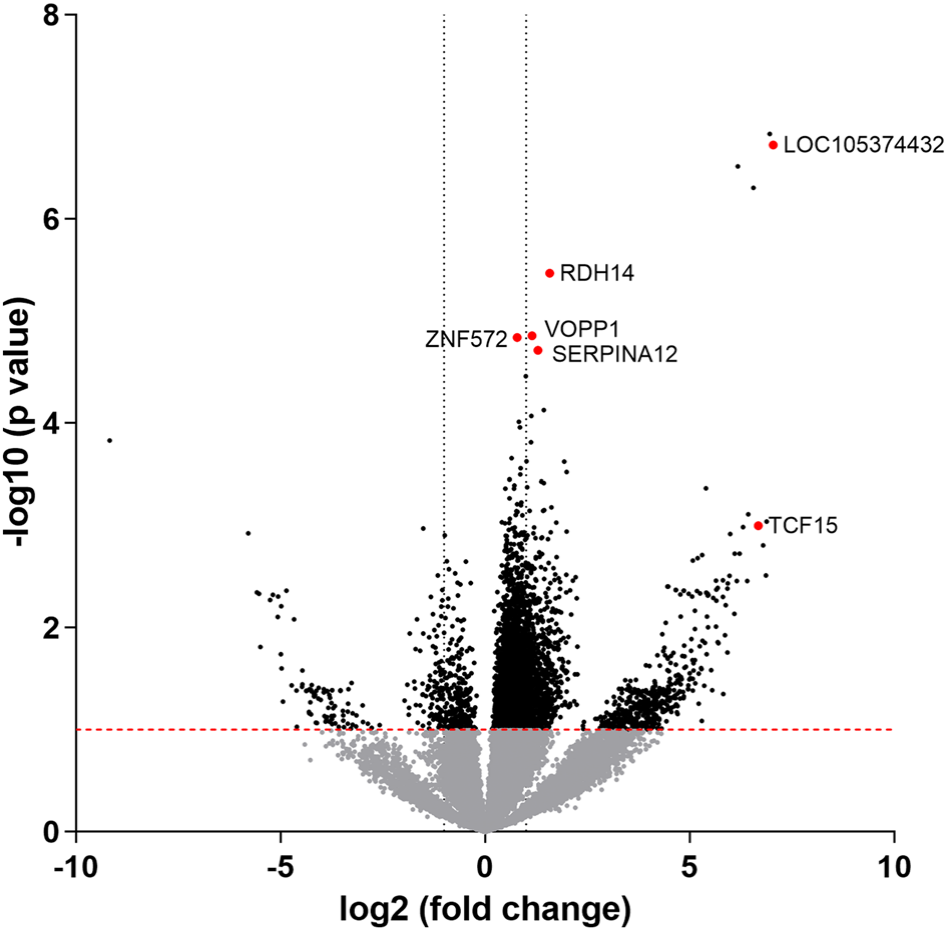
Volcano plot of sequencing data. Raw P-values in negative logarithmic scale on the y-axis as a function of the log2-fold change on the x-axis. Red dots represent the 6 upregulated candidate genes.

The candidate markers were miRNAs, SNORA, and uncharacterized genes (LOC105374432), including the ordinary genes. To overview statistical significance along with differential gene expression, volcano plots were drawn, in which the magnitude of gene expression ratios and the significance of the difference in gene expression between pools were displayed on the x-axis and y-axis, respectively. Red dots indicate the six upregulated DEGs (Fig. 3).

### qRT-PCR based validation of differentially expressed genes in the extended samples

**Figure 4:**
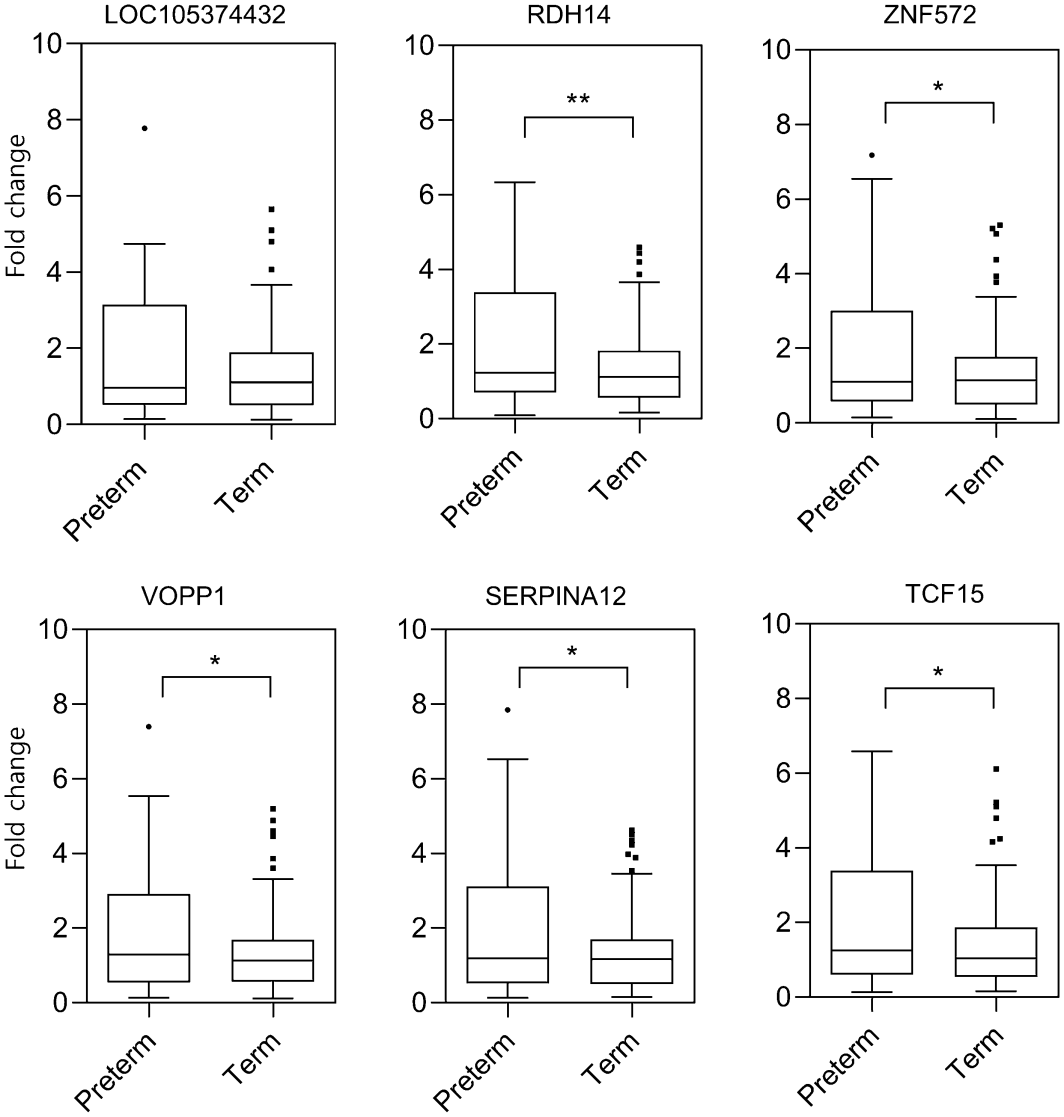
qRT-PCR validation of the expression of 6 chosen differentially expressed genes in the extended samples. Asterisks indicate levels of significance of differential expression (* P ≤ 0.05, ** P ≤ 0.01).

Common genes obtained from FC > 1.5 and FDR P-value < 0.1, such as MIR6801, SNORA108, MIR4749, are small RNA. However, in the case of small RNA, the PCR-based method for validation appears to be challenging because it requires a relatively large amount of starting material due to its small size. Consequently, the next top 6 upregulated DEGs (LOC105374432, RDH14, ZNF572, VOPP1, SERPINA12, and TCF15) were chosen from the ranked gene lists. For qRT-PCR based validation of the candidate genes, 21 s-trimester AFS samples from the preterm birth group and 40 from the term birth group, with an RNA quantity of more than 10 ng/ul were chosen. Consistent with the sequencing results, all 5 DEGs (RDH14, ZNF572, VOPP1, SERPINA12, and TCF15) were found to be differentially expressed in the preterm birth samples with the same trends (P < 0.05; Fig. 4 and Supplementary Table 3).

### Validation of candidate genes in H_2_O_2_-treated human trophoblast cell line HTR-8/SVneo and JEG-3 using qRT-PCR

**Figure 5:**
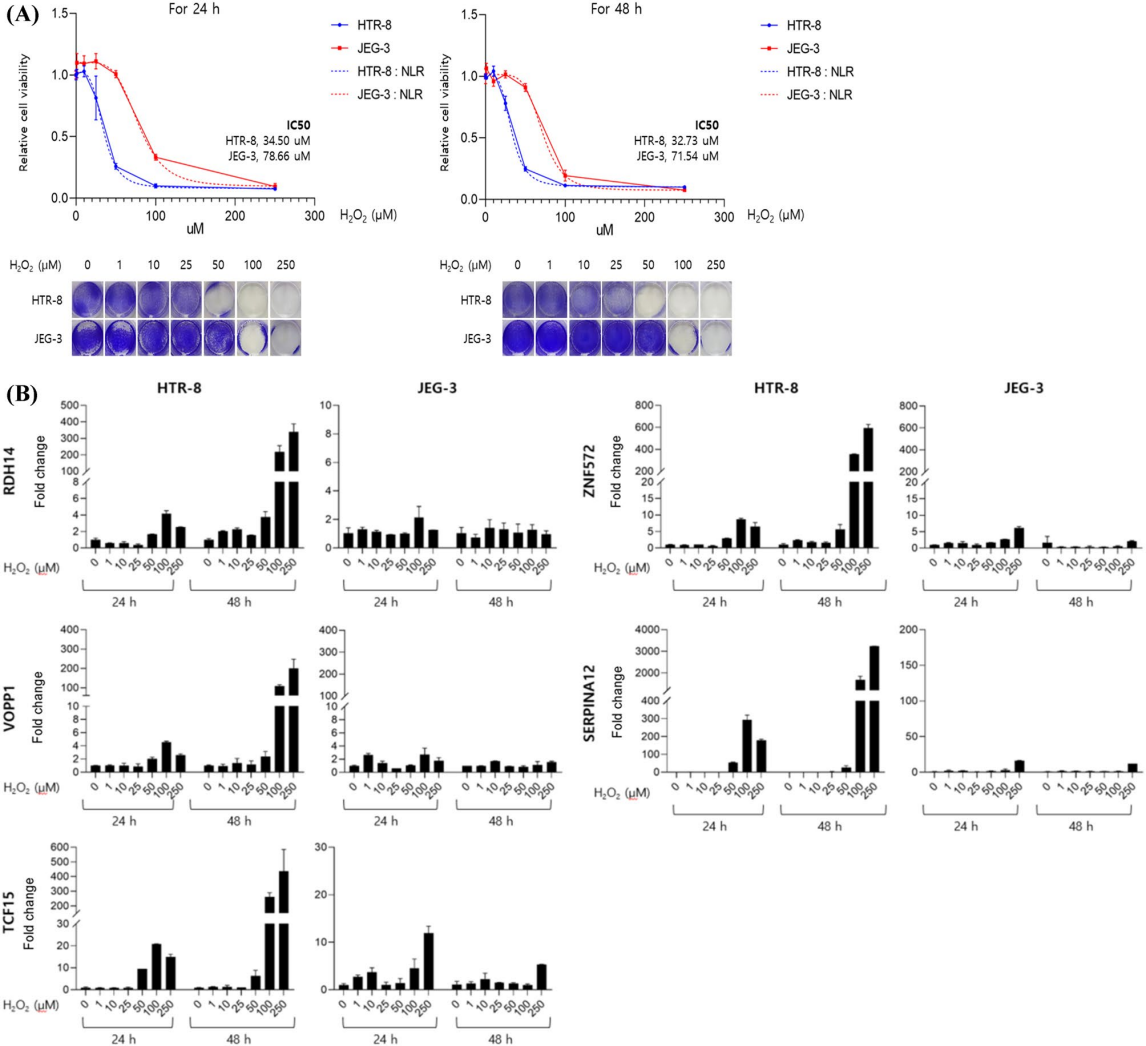
Validation of candidate genes in H_2_O_2_ treated human trophoblast cell line HTR-8/SVneo and JEG-3 cell lines. **(A)** Time- and dose-dependent cytotoxic activity in the HTR-8/SVneo and JEG-3 cell lines **(B)** qRT-PCR validation of the expression of 5 chosen differentially expressed genes in H_2_O_2_ treated human trophoblast cell line HTR-8/SVneo and JEG-3 cell lines.

A high level of systemic oxidative stress in the placenta is known to be associated with preterm birth. To explore the association between oxidative stress and candidate gene expression, we further compared the mRNA expression of candidate genes in H_2_O_2_-treated human trophoblast cell line HTR-8/SVneo and choriocarcinoma JEG-3. First, to compare the cytotoxic effects of H_2_O_2_ in each cell line, we performed a cell viability assay following treatment with different concentrations of H_2_O_2_ for 24 h and 48 h. As shown in Fig. 5-A, H_2_O_2_ significantly inhibited the viability of HTR-8/SVneo cells at concentrations of 25, 50, 100, and 250 μM after 24 h (P < 0.05), and the inhibitory effect of H_2_O_2_ on both cells was dose-dependent. HTR-8 cells were more sensitive than JEG-3 cell lines to H_2_O_2_ cytotoxicity. The IC50 values of HTR-8/SVneo and JEG-3 cells were 34.50 and 78.66 μM after 24 h and 32.73 and 71.54 μM after 48 h of treatment, respectively. Next, we performed expression analyses of RDH14, ZNF572, VOPP1, SERPINA12, and TCF15 using qRT-PCR analysis in the RNA samples extracted from two cell lines with H_2_O_2_ treatment to validate the candidate genes. Five candidate genes displayed a significant increase in mRNA expression in the HTR-8/SVneo cells after H_2_O_2_ treatment, but no change in the JEG-3 cells due to resistance to H_2_O_2_ treatment (Fig. 5B).

### Effect of liposaccharide treatment and glucose deprivation on the expression of candidate genes

**Figure 6:**
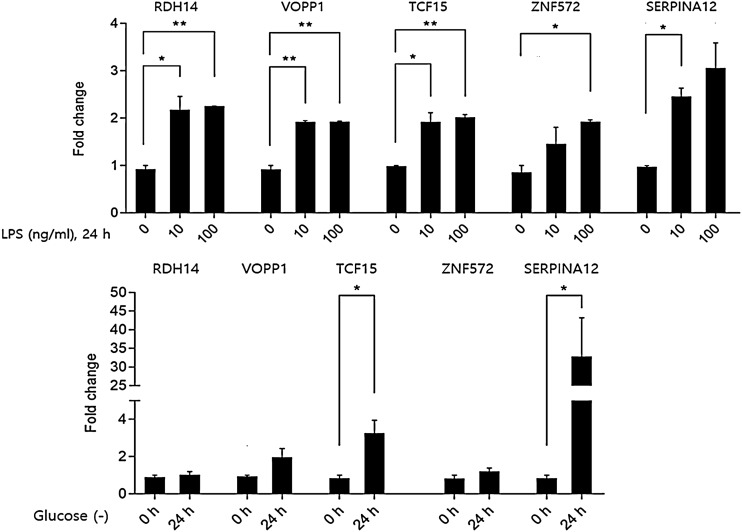
Effect of liposaccharide and glucose depriviation on the expression of the candidate genes in HTR-8/SVneo cells. Asterisks indicate levels of significance of differential expression (* P ≤ 0.05, ** P ≤ 0.01).

It has been shown that lipopolysaccharide (LPS)-induced oxidative stress is associated with preterm labor^25,26^ and glucose deprivation results in oxidative stress^27,28^. Thus, to examine the effect of LPS treatment and glucose deprivation on the expression of genes, we analyzed the expression of five candidate genes in glucose-deprived, LPS-induced HTR-8/SVneo cells using qRT-PCR analysis. The mRNA expression of all five candidate genes, RDH14, VOPP1, TCF15, ZNF572, and SERPINA12, were significantly increased in HTR-8/SVneo cells pretreated with LPS at a concentration of 10 ng/ml and 100 ng/ml. The expression of five candidate genes was increased more than twofold by pretreatment with LPS in HTR-8/SVneo cells. It was observed that the expression levels of TCF15 and SERPINA12 were upregulated in HTR-8/SVneo cells under glucose deprivation conditions in comparison with the control cells growing with glucose. Thus, LPS treatment and glucose deprivation led to the upregulation of candidate genes (Fig. 6).

### Effect of ritodrine on the cell viability and expression of candidate genes in H_2_O_2_-treated human trophoblast cell line HTR-8/SVneo

**Figure 7:**
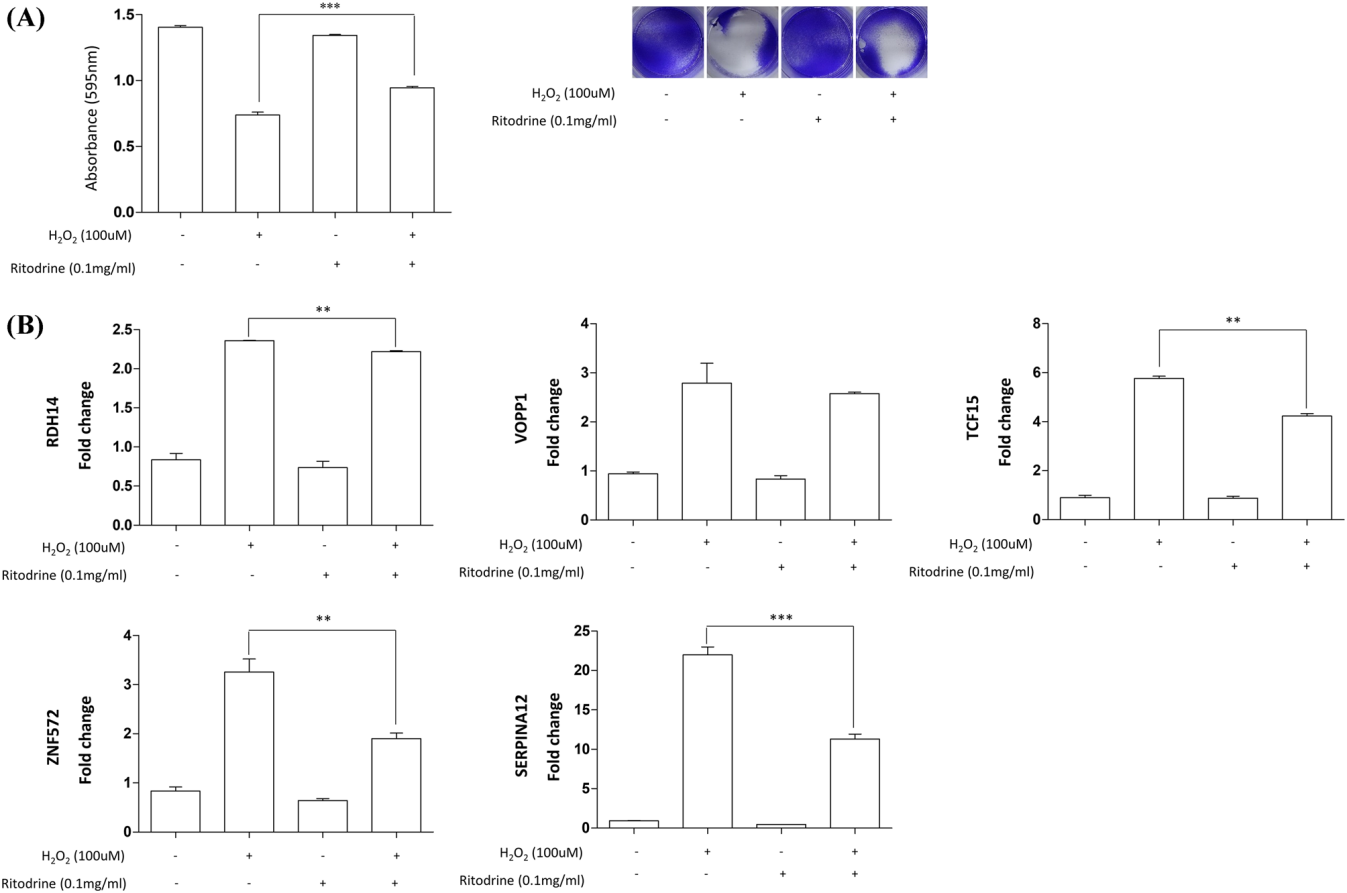
Effect of ritodrine on the cell viability **(A)** and mRNA expression of candidate genes **(B)** in H_2_O_2_-treated HTR-8/SVneo cells. Asterisks indicate levels of significance of differential expression (** P ≤ 0.01, *** P ≤ 0.001).

To evaluate the effect of ritodrine on H_2_O_2_-induced cell damage, we performed a cell viability assay following the treatment of 0 μM and 100 μM H_2_O_2_ with or without ritodrine for 24 h. As shown in Fig. 7A, the viability of cells treated with ritodrine alone without H_2_O_2_ was similar to that of the control group. Ritodrine significantly reversed the damaging effects of H_2_O_2_ on HTR-8/SVneo cells (P < 0.001), which indicated that ritodrine may exhibit potent protective potential against H_2_O_2_-induced oxidative damage. Furthermore, ritodrine significantly decreased the mRNA expression levels of RDH14, TCF15, ZNF572, and SERPINA12, which are normally increased by H_2_O_2_ in HTR-8/SVneo cells (Fig. 7B). Taken together, our results suggest that ritodrine protects against H_2_O_2_-induced cell damage by decreasing the expression of candidate genes, thereby inhibiting reactive oxygen species (ROS) generation.

## Discussion

This is the study comparing gene expression in the AFS between spontaneous preterm and term births using NGS. In this study, we analyzed the differences in global gene expression in the AF-derived cfNAs before the onset of preterm birth to identify the pathogenesis of spontaneous preterm birth. It is well known that AF is a complex and dynamic solution that can provide valuable information about fetal development and health status, and many studies have therefore reported the presence of cfNAs in AF. Although numerous studies have reported the use of AF cfNAs for discovering novel biomarkers for pregnancy complications, there have been no studies using AF-derived cfNAs to explore the pathogenesis of preterm birth. While other researchers have reported gene expression via microarray in AFS, few studies have performed gene expression analyses using sequencing analysis from AFS.

In this study, 30 DEGs were identified in the preterm birth cases compared with the term birth using nucleic acid sequencing. In particular, the expression patterns of 5 DEGs were verified by qRT-PCR in the second-trimester AFS of the preterm birth group and were highly associated with oxidative stress in the pathophysiology of preterm birth. Most DEGs were not deduced a valid ontology for each gene and particularly oxidative stress-related ontology because they were rarely studied on their functions. However, previous studies have shown that our candidate genes have already been correlated with oxidative stress in various experimental conditions and diseases. For example, the cardiotoxin-mediated transient acute mouse model of muscle degeneration lowered glutathione's reduction/oxidation rate as an oxidative stress marker and changed the protein expression level of RDH14^29,30^. The knock-down of VOPP1 overexpressed in squamous carcinoma cells induced cell death by increasing ROS and mitochondrial dysfunction, and N-acetyl cysteine as a ROS scavenger abolished the induction of apoptosis by VOPP1 knock-down^31^. In addition, SERPINA12 (Vaspin) inhibited apoptosis in human hepatocellular carcinoma cells, associated with reduced nitric oxide and superoxide anion^32,33^. Therefore, these results suggested that our candidate genes can be correlated with oxidative stress stimuli induced preterm birth.

Oxidative stress, characterized by imbalances in the redox system in the maternal–fetal intrauterine compartments, has been reported to play a critical role in the pathogenesis of spontaneous preterm birth. The present study showed that even before the onset of symptoms of preterm birth, there is a possibility of a redox imbalance in the maternal–fetal intrauterine compartments. Vora et al. examined the differences in gene expression in the cfRNA from AFS in women who delivered preterm and those who delivered at term using a customized nanostring panel containing genes related to oxidative stress^34^. They reported that changes in selected gene expression related to oxidative stress and inflammation occur before the onset of preterm birth, which may facilitate early detection of pregnancies at higher risk of preterm birth.

The role of inflammation related to the pathophysiology of preterm birth has been well studied, and several investigators have reported various inflammatory biomarkers associated with preterm birth. Oxidative stress, characterized by the generation of ROS, is an integral factor in the inflammatory process^35^. Recent studies have demonstrated that oxidative stress plays an important role in infection-induced apoptosis and in the pathogenesis of preterm labor^36,37^. LPS triggers inflammatory responses in human gestational tissues by increasing pro-inflammatory cytokines and phospholipid metabolites mediated by activation of the NF-κB pathway. In addition, glucose deprivation results in oxidative stress, and the alteration of the redox status of cells triggers stress-activated or other signal transduction pathways leading to cell death. In the present study, the expression of candidate genes was increased under conditions of oxidative stress and ROS production, which was induced by H_2_O_2_, LPS treatment, and glucose deprivation.

The production of ROS is necessary for the regulation of aerobic energy production. Redox balance is maintained through an intricate equilibrium between ROS production and subsequent elimination, and an interactive network of enzymatic and non-enzymatic antioxidant systems. Cells can protect themselves against oxidative stress by increasing the levels of endogenous and exogenous antioxidants or decreasing the production of ROS^38–40^. The balance between ROS and antioxidants can maintain good maternal health and safe childbirth^41,42^. Redox imbalance is closely related to the fundamental pathogenesis of many pregnancy complications. The overproduction of ROS in inflammation and cell damage can generate pathways leading to preterm birth. The risk factors for preterm birth can lead to redox imbalance caused by high levels of ROS, such as hydroxyl radicals, hydrogen peroxide, superoxide anion, and nitric oxide. As a result, ROS-mediated oxidative stress contributes to collagen degradation and consume antioxidant defenses. Collagen damage in human gestational tissue can result in uterine contractions and tears in the chorioamniotic sac, leading to spontaneous preterm birth.

This study showed that ritodrine, a type of β_2_-adrenoreceptor agonist used as a treatment for preterm labor, decreased the expression of candidate genes, thereby inhibiting H_2_O_2_-induced cell damage. β_2_-adrenoreceptor agonists have been considered to have anti-inflammatory and antioxidant properties, which can be of great interest in treating several diseases^43,44^. However, the exact mechanism for decreasing oxidant production of inflammatory cells remains unknown. In previous studies, low doses of β_2_-adrenoreceptor agonist significantly inhibited LPS-induced activation of NF-kB and production of proinflammatory cytokines normally mediated by the NF-kB and MAPK signaling pathway^43^. Also, these studies showed reduced ROS generation and decreased apoptosis after β-adrenergic receptor stimulation, which support our findings that demonstrate that β_2_-adrenergic receptor stimulation increases the cell viability. Our results suggest that β_2_-adrenergic receptors stimulation inhibits the activation of candidate genes, supporting reduced apoptosis and low level of ROS after β_2_-adrenergic receptor stimulation.

Cell-free fetal nucleic acids (cff NAs) have become the subject of intense interest in prenatal diagnosis since Lo et al. found their presence in maternal blood in 1997^45^. Cff NAs in maternal plasma are now established as molecular diagnostic materials for noninvasive prenatal testing and have been routinely used in clinical care. Through advancements in sequencing technology and large scale genomic profiling in both clinical and research fields, simple methods to generate comprehensive and accurate whole-genome and transcriptome sequencing data will become increasingly valuable. Until now, RNA-sequencing analysis provides detailed information of the transcriptome while enabling a novel RNA transcript variation to be detected. Recently, the integration of DNA and RNA analysis has shown promising results in the detection of fusion transcripts and alternative transcripts. These sequencing data can provide useful information to explore genetic variation underlying human disease and normal phenotypic variability^46^.

Nucleic acid sequencing is an advanced technology for genome analysis that enables a comprehensive characterization of gene expression. Most gene expression data regarding human pregnancy are based on microarrays^47–49^, and most of these are focused on the transcriptome of preeclampsia, not on preterm birth. In addition, published studies on human labor using NGS have been confined to the placenta or blood. Until now, many studies have focused on maternal factors, such as inflammation, infection, and maternal gestational tissues, in studying the causes of preterm birth; however, little is known about the fetal contribution to preterm birth^36,50–52^. Although maternal factors are known to contribute to preterm birth risk, there are emerging data that variations in the fetal gene expression, not the maternal, may induce preterm labor^34,53^. If sequencing technologies can analyze the DEGs in preterm birth to identify unique gene signatures at the fetal molecular level, it will help to understand more about the fetal contribution to pregnancy-specific diseases such as preterm birth. Further, it will lay the groundwork for preventive, diagnostic, and therapeutic strategies in obstetrics and pediatrics. Overall, both maternal and fetal genomes can simultaneously or separately contribute to the occurrence of spontaneous preterm birth, influenced by the environmental factors.

We aimed to investigate gene expression signatures that contribute to preterm birth prior to the onset of symptoms of preterm birth. Based on these results, we identified the difference in gene expression associated with redox imbalance as a contributing factor to preterm birth. This led to further investigations to define high-risk populations and characterize gene expression in the AFS. However, there are several limitations in this study. First, we performed a comparative study with sequencing data obtained from five samples each of preterm and term births. This relatively small sample size makes it difficult to draw definitive conclusions. While it is widely acknowledged that increasing the number of replicates in sequencing experiments usually results in more reliable results, the exact relationship between the number of replicates and the possibility of identifying DEGs has not been thoroughly investigated. However, Conesa et al. recommended that sequencing experiments have at least three biological replicates if sample availability is not limited to allow all sequencing analysis to utilize reproducibility between replicates^54^. Second, this study may have annotation biases because of inequality in gene annotations of bioinformatics analysis. Third, it lacks sufficient power to demonstrate the mechanisms leading to early delivery due to the lack of early preterm births in our study. The preterm birth group in our study included only late preterm births (34–36 weeks), and there was only a difference of about 5 weeks compared to the mean gestational age at delivery of the term birth group. The absence of an early primordial group in sample collection resulted in a limitation, but it is still another significance of this study that gene expression differences occurred in second-trimester AF prior to 20–25 weeks before delivery.

Despite these limitations, this study has many strengths. This study investigated differences in gene expression levels using cfNAs isolated from AFS in the early second-trimester of gestation for the prediction of spontaneous preterm birth. This information regarding the variations occurring at the genome or transcriptome level will be help in improved antenatal recognition, thereby reducing the morbidity and mortality associated with preterm birth. Identification of differences in gene expression between preterm birth and term birth before the onset of clinical symptoms will lay the groundwork for future prospective studies to delineate mechanisms leading to preterm birth.

In conclusion, specific expression patterns of genes associated with high oxidative stress in pregnant women may indicate placental tissue damage predisposing to spontaneous preterm birth. Expression patterns of candidate genes shown in our study may be a criterion for assigning therapeutic targets so as to regulate their function at the earliest, thereby modulating the expression of proteins and their pathogenic pathways in spontaneous preterm birth. These DEGs may elucidate the underlying mechanisms of preterm birth and be used to predict preterm birth in early pregnancy.

## Materials and methods

### Patients and amniotic fluid collection

AF samples were obtained from 74 pregnant women who underwent amniocentesis for fetal karyotyping and genetic diagnosis at 16 to 19 weeks of gestation in the Department of Obstetrics and Gynecology, Gangnam Severance Hospital (Seoul, Korea) between March 2011 and May 2017. This study was reviewed and approved by the Institutional Review Board of Gangnam Severance Hospital (Ethics ref.: 3-2011-0147). All participants provided written informed consent to participate in the study before AF was obtained. All experiments were performed in accordance with the relevant guidelines and regulations. For all subjects, gestational age was assessed during early gestation using the crown-rump length measurement by transvaginal ultrasonography. We included only cases of spontaneous preterm birth and excluded all pregnancies complicated by preeclampsia, major fetal anomalies, abnormal karyotypes, gestational diabetes mellitus and multiple pregnancy. We defined control subjects as those who had normal term births, defined as a live birth from 37 to 42 weeks of gestation.

### Cell-free circulating nucleic acid extraction

AF samples were centrifuged at 350 × *g* for 10 min at 4 °C. The supernatant samples were spun at 1500 rpm for 10 min at 10 °C to remove residual vernix and then stored at -80 °C. CfNAs were extracted from 1 mL of AFS using the QIAamp Circulating Nucleic Acid kit (Qiagen, Germany) according to the manufacturer's protocol and eluted with RNase-free water. The concentration of cfNAs in each sample was measured using a NanoDrop ND-1000 spectrophotometer (Nanodrop Technologies Inc., Wilmington, DE, USA). Samples with an A260/A280 ratio greater than 2.0 were stored at −70 °C until further analyses.

### Sequencing analysis

The quantity and quality of the extracted nucleic acids were assessed using an Agilent 2100 Bioanalyzer RNA chip (Agilent Technologies, Santa Clara, CA, USA) according to the manufacturer’s instructions. Sequencing libraries were prepared using the SMARTer Stranded Total RNA-Seq kit-v2 Pico Input Mammalian (Takara Bio USA, Mountain View, CA, USA) and validated using a DNA 1000 chip on an Agilent 2100 Bioanalyzer (Agilent Technologies) according to the manufacturer’s instructions. Nucleic acid sequencing was performed using the Illumina NextSeq 500 platform (Illumina, Inc., San Diego, CA, USA) with a 75-nucleotide paired-end indexed run.

### Sequencing read mapping and gene expression signature analysis

Nucleic acid sequencing reads were mapped to the human genome reference (Homo sapiens GRCh38 dbSNP150genome template, obtained from DNASTAR, Inc., Madison, USA). The read counts for each gene were calculated and analyzed using three different normalizations, that is, quantile, RPM, and RPKM, and non-normalization methods for differential expression detection. Gene transcript numbers comparing preterm birth versus term birth were considered to be significantly different by using a combination of FC > 1.5 and P-value (FDR P-value < 0.1 or P-value < 0.01)^55^. Significant differences in gene expression were determined via Student's t-test. DEGs were retained if they were detected by at least two of the methods used. All analyses were performed using DNASTAR Lasergene 15 software. Common markers were identified using a heat map with hierarchical clustering and Venn Diagram analysis.

### Cell viability assay

JEG-3 and HTR-8/SVneo cell lines were plated onto 24-well plates at densities of approximately 8.0 × 10^4^ cells and 1.0 × 10^5^ cells per well, respectively, in 0.5 ml of growth media. After 24 h, the medium was replaced with DMEM containing 10% FBS. Meanwhile, H_2_O_2_ (0, 1, 10, 25, 50, 100, and 250 μM) was added to the corresponding wells and incubated at 37 °C for 24 h or 48 h. For the crystal violet staining assay, cells were fixed with fixation solution (10% acetic acid, 10% methanol, 80% H_2_O),stained with 0.5% crystal violet staining solution with 20% methanol for 1 h, photographed, and extracted using 1% SDS solution. The absorbance was measured at 595 nm using a VersaMax microplate reader (Molecular Devices, Sunnyvale, CA). All experiments were performed in triplicates. We investigated the effect of ritodrine (Lavopa®; JW Pharmaceutical, Seoul, Korea), a type of β_2_-adrenoreceptor agonist used as a treatment for preterm labor, on H_2_O_2_-induced cell damage. HTR-8/SVneo cells were seeded onto 24-well plates at a density of approximately 2.0 × 10^5^ cells per well in 0.5 ml of growth media. After 24 h, the medium was replaced with DMEM containing 10% FBS. Meanwhile, cells were treated with 0.1 mg/ml of ritodrine; 30 min later, 0 μM or 100 μM H_2_O_2_ was added to the plate and incubated at 37 °C for 24 h.

### Lipopolysaccharide treatment and glucose deprivation

HTR-8/SVneo cells were seeded at 4.0 × 10^5^ cells in six well plates and treated with 10 ng/ml and 100 ng/ml LPS (Sigma-Aldrich, St. Louis, MO, cat. L4391) for 24 h. For glucose deprivation, the complete DMEM medium in culture plates was replaced with glucose-free DMEM medium (Thermo Fisher Scientific, Rockford, IL, USA, cat. 11,966–025) and cells were cultured for 24 h.

### Quantitative real-time polymerase chain reaction

Total RNA (50 ng) from each sample was reverse-transcribed into cDNA using Maxima First Strand cDNA Synthesis Kit (Thermo Scientific, Waltham, MA) according to the manufacturer’s protocol. Real-time polymerase chain reaction (PCR) was performed to quantify mRNA expression using SYBR Green PCR Master Mix (Enzynomics, Daejeon, Republic of Korea) and an ABI PRISM 7300 real-time PCR system (Applied Biosystems, Foster City, CA) according to the manufacturer’s instructions. Relative mRNA expression was quantified using the comparative Ct (ΔCt) method and expressed as 2-ΔΔCt, where ΔΔCt = ΔE–ΔC, ΔE = CtE target–CtE GAPDH, and ΔC = CtC target–CtC GAPDH (E = experimental result and C = controls). Each assay was performed in triplicate and expressed as the mean ± standard error (SE). Serial dilutions were prepared from a stock solution of total RNA to generate a standard curve and determine reaction efficiencies. The primers used for PCR are listed in Supplementary Table 4.

### Statistical analysis

Experimental results were statistically evaluated with a two-tailed paired Student’s t-test using GraphPad Prism 9. All tests of significance were set at P < 0.05.

## Supplementary Information

Supplementary Tables.

Supplementary Figure 1.
