# Supplementary material for: Identification of a novel gene signature in second-trimester amniotic fluid for the prediction of preterm birth
Source: Sci Rep. 2022 Mar 31;12:3085. doi: 10.1038/s41598-021-04709-3 (PMC8971495; doi:10.1038/s41598-021-04709-3)
Supplement: Supplementary file 1 — Supplementary Tables. [file 41598_2021_4709_MOESM1_ESM.pdf]

Supplementary Table 1. Clinical characteristics of the study population

Sample	Age (years)	GA at amniocentesis (weeks)	Indication of amniocentesis	GA at delivery (weeks)	Pre-pregnancy BMI (kg/m^2)	PPROM	Smoking
P1	39	16+3	AMA	35+0	26.7	Yes	No
P2	37	17+2	AMA	35+4	25.5	Yes	No
P3	33	16+5	Positive screening test for Down syndrome	36+6	20.4	No	No
P4	35	17+3	Positive screening test for Down syndrome	36+3	18.9	No	No
P5	32	18+4	Positive screening test for Down syndrome	36+4	22.7	No	No
N1	31	16+2	Positive screening test for Down syndrome	40+4	22.4	-	No
N2	35	17+2	Positive screening test for Down syndrome	40+1	16.9	-	No
N3	39	16+0	AMA	40+5	20.8	-	No
N4	41	18+2	AMA	40+0	18.4	-	No
N5	39	17+4	Positive screening test for Down syndrome	40+0	19.8	-	No

GA, gestational age; AMA, advanced maternal age; BMI, body mass index; PPROM, preterm premature rupture of membranes

In sample names, the prefix P is used for preterm birth and N for normal term birth.

Supplementary Table 2. The candidate gene markers for the preterm birth

Name	Non-normalization					Name	Quantile normalization					Name	RPM normalization					Name	RPKM normalization				
	Preterm Birth	Term Birth	FDR P-value	P-value	Fold change		Preterm Birth	Term Birth	FDR P-value	P-value	Fold change		Preterm Birth	Term Birth	FDR P-value	P-value	Fold change		Preterm Birth	Term Birth	FDR P-value	P-value	Fold change
**Sorting by Fold change >1.5 and FDR p < 0.1**																							
MIR6801	2.605	0.021	0.00357	1.47E-07	125.048 up	MIR4420	0.084	1.326	0.00824	2.17E-07	15.836 down	MIR6801	1.399	0.009	0.00215	9.94E-08	163.579 up	MIR6801	62.539	0.024	0.000146	3.84E-09	2582.261 up
LOC105374432	2.759	0.021	0.00357	1.88E-07	132.454 up							SNORA108	0.814	0.009	0.00215	1.13E-07	95.172 up	SNORA108	6.119	0.024	0.000438	2.45E-08	252.649 up
SNORA108	1.516	0.021	0.00387	3.05E-07	72.754 up							LOC105374432	1.482	0.009	0.00383	3.02E-07	173.267 up	MIR4749	49.434	0.024	0.000438	3.45E-08	2041.176 up
MIR4749	1.974	0.021	0.00471	4.95E-07	94.768 up							MIR4749	1.06	0.009	0.00846	8.89E-07	123.969 up	LOC105374432	6.704	0.024	0.00146	1.54E-07	276.811 up
**Sorting by Fold change >1.5 and p < 0.01**																							
RDH14	29.913	10.005	0.026	3.41E-06	2.989 up	ARHGAP19	0.153	31.974	0.407	0.0000321	209.073	ARHGAP19	0.021	17.484	0.68	0.0000893	826.915	ARHGAP19	0.045	12.01	0.215	0.0000282	265.690
ARHGAP19	0.052	30.044	0.39	0.000148	578.813	RDH14	27.736	11.153	0.856	0.000235	2.486 up	RDH14	16.062	5.822	0.681	0.000108	2.758 up	RDH14	15.886	5.758	0.68	0.000108	2.759 up
GNB4	21.803	10.027	0.39	0.000154	2.174 up	GNB4	19.689	11.808	0.856	0.000263	1.667 up	LOC102723321	17.767	40.544	0.874	0.000161	2.282 down	LOC102723321	13.923	31.767	0.876	0.000161	2.281 down
LOC107984025	9.509	2.491	0.505	0.000239	3.816 up	LOC102723321	30.668	73.252	0.938	0.000561	2.388 down	LOC107987205	2.724	0.02	0.953	0.00041	137.366 up	CHRM5	17.946	10.813	0.961	0.000786	1.659 up
LOC101927474	19.199	7.039	0.643	0.000703	2.727 up	TMEM225	26.611	12.151	0.938	0.000906	2.189 up	IGHMBP2	49.415	28.428	0.953	0.000505	1.738 up	IGHMBP2	17.229	10.172	0.961	0.000902	1.693 up
TMEM225	28.903	10.807	0.643	0.000719	2.674 up	IGHMBP2	88.784	52.238	0.938	0.00098	1.699 up	CHRM5	28.698	17.294	0.953	0.000783	1.659 up	RCAN1	42.808	21.374	0.961	0.000934	2.002 up
IGHMBP2	92.028	48.848	0.643	0.00079	1.883 up	SPATA16	11.873	34.209	0.938	0.00121	2.881 down	TMEM225	15.52	6.289	0.953	0.000978	2.467 up	TCF15	4.747	0.058	0.961	0.00103	81.812 up
LOC107987205	6.128	0.052	0.643	0.000916	118.056 up	MROH5	54.677	32.883	0.938	0.00127	1.662 up	RCAN1	25.769	12.282	0.953	0.00105	2.098 up	TMEM225	29.893	11.961	0.961	0.00132	2.499 up
LOC105376196	16.027	4.789	0.643	0.000936	3.346 up	TCF15	4.461	0.225	0.938	0.00139	19.786 up	TCF15	40.568	26.901	0.953	0.00106	1.508 up	LOC107984025	12.244	3.476	0.961	0.00157	3.522 up
TCF15	5.305	0.052	0.643	0.001	102.196 up	LOC107986385	0.21	4.739	0.938	0.00141	22.540 down	LOC107984025	2.848	0.023	0.953	0.00118	125.042 up	LOC107986124	7.792	15.804	0.961	0.00197	2.028 down
SEMA6B	37.363	12.309	0.643	0.00116	3.035 up	LOC105376196	14.452	5.989	0.938	0.0015	2.413 up	UGT2A3	55.543	37.744	0.953	0.00139	1.471 up	LOC107987205	4.463	0.062	0.961	0.00215	71.689 up
MROH5	57.67	29.829	0.643	0.00116	1.933 up	LOC107986124	50.746	105.374	0.938	0.00157	2.076 down	LOC107986124	5.106	1.45	0.953	0.00158	3.521 up	LLGL1	10.155	5.962	0.961	0.0023	1.703 up
RCAN1	47.99	21.104	0.643	0.00119	2.274 up	UGT2A3	17.084	41.388	0.938	0.00194	2.422 down	LLGL1	10.113	22.263	0.953	0.00177	2.201 down	SEMA6B	8.134	2.96	0.961	0.00294	2.748 up
LLGL1	61.562	33.407	0.643	0.0015	1.842 up	LOC105373924	3.632	0.288	0.938	0.00209	12.600 up	AREL1	16.907	8.688	0.953	0.0018	1.946 up	AREL1	14.816	9.156	0.961	0.0031	1.618 up
AREL1	64.596	36.84	0.643	0.00219	1.753 up	AREL1	61.691	39.648	0.938	0.00215	1.555 up	SEMA6B	28.847	58.52	0.953	0.00197	2.028 down	GNB4	11.443	5.703	0.961	0.00314	2.006 up
LOC105377527	37.925	12.953	0.643	0.00233	2.927 up	SEMA6B	34.721	14.059	0.938	0.00216	2.469 up	GNB4	33.056	19.442	0.953	0.00228	1.700 up	UGT2A3	3.414	7.35	0.961	0.00318	2.153 down
LOC105376339	17.049	5.346	0.643	0.00262	3.188 up	CHRM5	50.497	31.926	0.938	0.00256	1.581 up	SPATA16	34.685	21.44	0.953	0.0029	1.617 up	LOC101927474	6.689	2.658	0.961	0.00384	2.516 up
SLC5A2	23.883	7.922	0.643	0.00366	3.014 up	LOC107987205	4.363	0.225	0.938	0.00274	19.352 up	LOC101927474	29.277	18.479	0.953	0.00308	1.584 up	SPATA16	4.374	12.132	0.961	0.00394	2.773 down
ABCA7	96.441	45.702	0.643	0.00395	2.110 up	LOC105376339	15.424	6.461	0.938	0.00293	2.387 up	LOC105377527	20.062	7.163	0.953	0.00309	2.800 up	LOC107986385	0.116	5.348	0.961	0.00457	45.959 down
CHRM5	53.446	29.717	0.643	0.00436	1.798 up	RCAN1	44.786	23.062	0.938	0.00303	1.941 up	LOC107986385	11.707	5.836	0.953	0.00314	2.006 up	LOC105377527	10.589	3.919	0.961	0.00491	2.701 up
LOC105373924	4.258	0.098	0.643	0.00465	43.448 up	LLGL1	58.293	36.014	0.938	0.00318	1.618 up	SLC5A2	7.152	18.795	0.953	0.00366	2.627 down	LOC105373924	5.036	0.124	0.961	0.00646	40.539 up
LOC107986385	0.098	4.513	0.643	0.00467	46.047 down	ABCA7	93.675	49.486	0.938	0.00356	1.892 up	LOC105373924	10.309	4.096	0.953	0.00384	2.516 up	SLC5A2	6.252	2.271	0.961	0.00717	2.752 up
LOC107986124	53.723	100.555	0.643	0.00519	1.871 down	SLC5A2	21.972	9.627	0.938	0.00539	2.282 up	ABCA7	20.364	7.538	0.953	0.00492	2.701 up	ABCA7	9.091	4.675	0.961	0.00732	1.944 up
UGT2A3	18.834	38.973	0.643	0.00538	2.069 down	LOC107984025	8.44	3.113	0.938	0.00666	2.711 up	MROH5	0.047	2.626	0.953	0.00529	56.026 down	MROH5	8.019	4.488	0.961	0.00852	1.786 up
LOC102723321	33.088	69.667	0.65	0.00623	2.105 down	LOC101927474	17.451	8.102	0.938	0.00688	2.154 up	LOC105376339	12.824	4.61	0.953	0.00661	2.781 up	LOC105376339	16.059	5.457	0.961	0.00874	2.942 up
SPATA16	13.32	32.295	0.675	0.00739	2.424 down	LOC105377527	35.508	14.065	0.938	0.00714	2.524 up	LOC105376196	2.286	0.049	0.953	0.00712	47.140 up	LOC105376196	12.711	4.116	0.961	0.00984	3.088 up

Supplementary Table 3. Validation data of selected differentially expressed genes by qRT-PCR

		**LOC105374432**		**RDH14**		**ZNF572**	
		Preterm	Term	Preterm	Term	Preterm	Term
n		21	40	21	40	21	40
Mean		1.777	1.416	1.98	1.36	1.91	1.435
Std. Deviation		1.699	1.193	1.696	1.044	1.819	1.223
Std. Error		0.2622	0.1333	0.2617	0.1167	0.2807	0.1367
95% CI		1.247 to 2.306	1.150 - 1.681	1.451 to 2.508	1.128 to 1.592	1.343 to 2.477	1.163 to 1.707
unpaired t-test	two-tailed	0.175		0.014*		0.0889	
	one-tailed	0.0873		0.007**		0.0444*	

		**VOPP1**		**SERPINA12**		**TCF15**	
		Preterm	Term	Preterm	Term	Preterm	Term
n		21	40	21	40	21	40
Mean		1.941	1.394	1.963	1.391	2.079	1.437
Std. Deviation		1.778	1.157	1.868	1.13	1.801	1.287
Std. Error		0.2743	0.1293	0.2883	0.1264	0.2778	0.1449
95% CI		1.387 to 2.495	1.137 to 1.652	1.380 to 2.545	1.139 to 1.642	1.518 to 2.640	1.148 to 1.725
unpaired t-test	two-tailed	0.0425*		0.0373*		0.0252*	
	one-tailed	0.0213*		0.0187*		0.0126*	

Supplementary Table 4. Primer sequences for Quantitative Real-Time Polymerase Chain Reaction

**Marker name**	**Gene bank**	**PCR product size**	**Forward sequence**	**Reverse sequence**
RDH14	NC_000002.12	70bp	TCTTCTGAACAAATGCCCTCTGA	TCCGTTGAGATTGAGGTAGAAGAGT
SERPINA12	NC_000014.9	70bp	TCCATCTTCAGCTCAGCCTTGT	AGGAGCTTGGCAGACCTTGA
VOPP1	NC_000007.14	70bp	GAGTCTACGGGCCACGATTTAG	CACAGCGACCACCTGTTGTT
ZNF572	NC_000008.11	77bp	ATTCAGCAGCAGCTCTCACCTTA	CGCAGACAGAACATTCATATGGTT
TCF15	NC_000020.11	70bp	AGGTGATCCCATCTCTGATGCT	CCTTCAGGCAGGTAGTTTTTCTG
LOC105374432	NC_000004.12	80bp	CTCGTCTGTTCTCATGCTGCTAA	TGTGAGTCCATTAAACCTCTTTCCT
GAPDH	NM_001256799	292bp	CCTGACCTGCCGTCTAGAAA	GGTGGTCCAGGGGTCTTACT
