# Supplementary figures and images for: Identification of a novel gene signature in second-trimester amniotic fluid for the prediction of preterm birth
Source: Sci Rep. 2022 Mar 31;12:3085. doi: 10.1038/s41598-021-04709-3 (PMC8971495; doi:10.1038/s41598-021-04709-3)

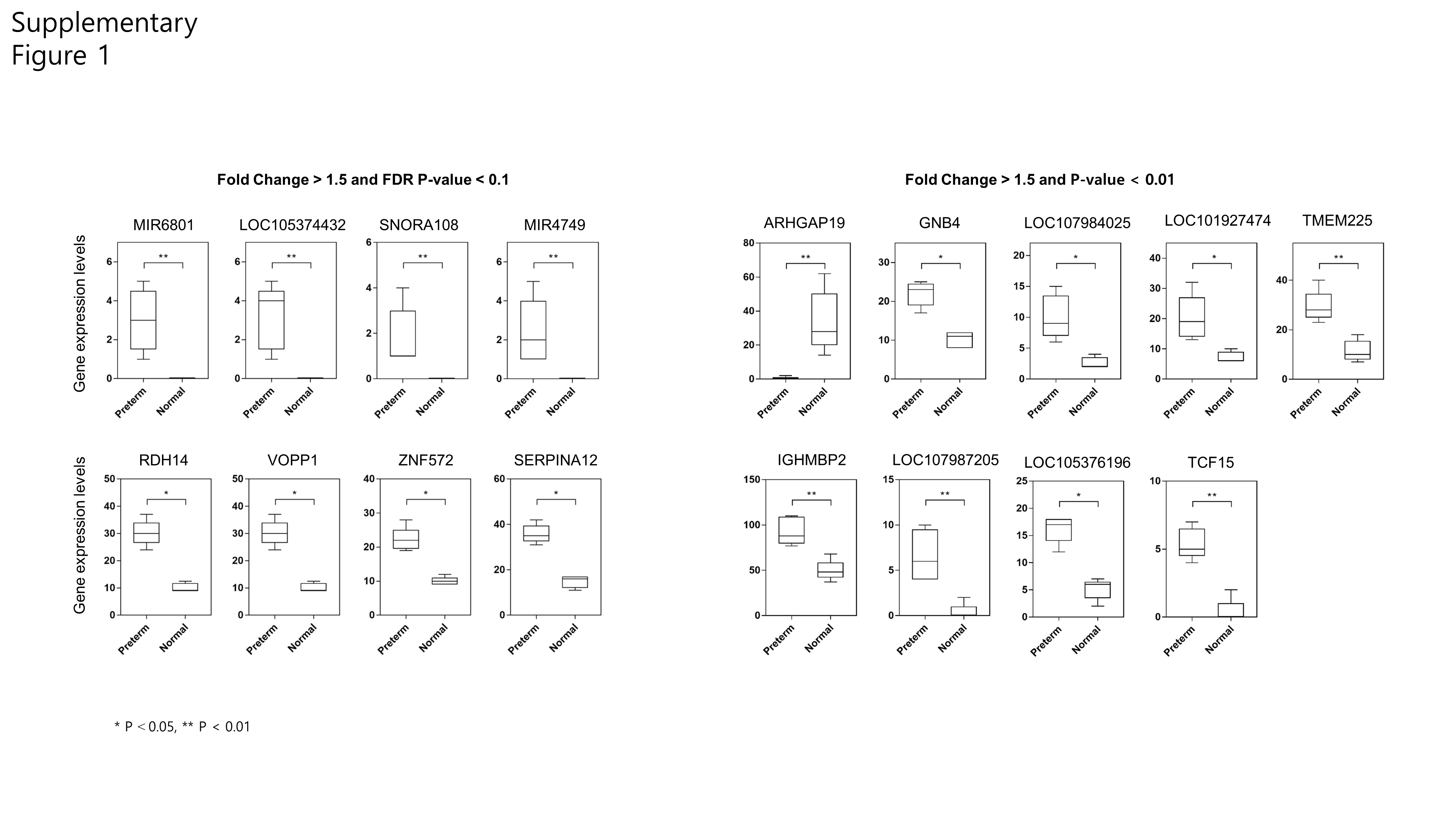

Supplement: Supplementary file 2 — Supplementary Figure 1. [file 41598_2021_4709_MOESM2_ESM.tif]
